# Early-stage macular telangiectasia type 2 vascular abnormalities are associated with interdigitation zone disruption

**DOI:** 10.1371/journal.pone.0259811

**Published:** 2021-11-12

**Authors:** Janice X. Ong, Roya Zandi, Amani A. Fawzi

**Affiliations:** Department of Ophthalmology, Feinberg School of Medicine, Northwestern University, Chicago, IL, United States of America; Singapore National Eye Centre, SINGAPORE

## Abstract

**Purpose:**

To investigate the relationship between disruption in different photoreceptor layers and deep capillary plexus (DCP) telangiectasias in eyes with macular telangiectasia type 2 (MacTel).

**Methods:**

35 eyes (21 patients) with MacTel imaged with optical coherence tomography angiography (OCTA) were included. Circumscribed areas of DCP telangiectasia were traced from OCTA slabs and the corresponding spectral-domain OCT (SD-OCT) slabs were used to visualize the photoreceptor layer interdigitation zone (IZ) and ellipsoid zone (EZ). IZ attenuation, IZ loss, and EZ loss were graded by reviewing *en face* SD-OCT slabs for hypo-reflective areas and confirming their status on cross-sectional views. Total area of photoreceptor disruption and overlap with DCP telangiectasia were evaluated with respect to OCT-based MacTel stage. Longitudinal changes were evaluated in a subset of patients with follow-up imaging.

**Results:**

Overlap of DCP telangiectasia with IZ attenuation significantly decreased with MacTel severity, while overlap with IZ and EZ loss significantly increased. Overlap with IZ loss peaked in moderate MacTel (Stages 3–5). Longitudinal imaging showed that new EZ loss at 6 months was largely predicted by baseline IZ loss.

**Conclusions:**

Worsening MacTel severity is characterized by greater overlap between DCP telangiectasia and zones of increasing severity of photoreceptor disruption, with EZ loss enlarging over time within areas of preexisting IZ disruption. We suggest that IZ disruption may indicate early photoreceptor dysfunction that eventually progresses to EZ loss, with IZ loss being a more reliable metric than IZ attenuation. Additional studies will be necessary to further explore long-term photoreceptor changes and evaluate their relationship with visual function in MacTel.

## Introduction

Idiopathic macular telangiectasia type 2 (MacTel) is a rare, bilateral retinal disease of unknown heritability that results in progressive vision loss [1]. Although a higher prevalence of systemic cardiometabolic conditions such as hypertension and diabetes mellitus has been associated with MacTel, the contribution of this association to the disease is unclear [2]. The pathophysiology of MacTel is thought to be primarily neurodegenerative, originating from the loss of Müller glia, which play a supportive and regulatory role to the photoreceptors and retinal vasculature [3–5]. Patients present with a variety of findings including juxtafoveal telangiectasias in the deep capillary plexus (DCP), right-angle venules, diminished retinal transparency, crystalline deposits, cavitary lesions, abnormal distribution of macular pigment, and photoreceptor atrophy [6–8]. Subretinal neovascularization is seen in more advanced disease and marks the transition from nonproliferative to proliferative MacTel [9].

The ellipsoid zone (EZ) is the second of four hyperreflective outer retinal bands seen on spectral-domain optical coherence tomography (SD-OCT) [10]. EZ loss is considered a marker of photoreceptor dysfunction and has been shown to correlate with disease severity and vision loss in MacTel [11–15]. Our group previously demonstrated that the percentage of overlap between DCP telangiectasia and EZ loss increased with advancing MacTel severity, with the greatest extent of overlap seen in proliferative disease [16]. Interestingly, we found that in non-proliferative MacTel eyes, DCP telangiectasia was localized to the borders of EZ loss, while the earliest stages of MacTel showed DCP telangiectasia without any evidence of EZ loss. Other studies have also suggested that areas of EZ loss on SD-OCT do not necessarily correlate with areas of photoreceptor abnormalities identified on other imaging modalities such as adaptive optics scanning laser ophthalmoscopy (AOSLO) [17, 18]. To explain these apparent discrepancies, we proposed that the margins of EZ loss might represent areas of subclinical photoreceptor dysfunction, or “photoreceptors at risk” [16].

The interdigitation zone (IZ), also called the cone outer segment tips, is the third hyperreflective outer retinal band on SD-OCT [10]. It is thought to represent the contacts of cone outer segments with specialized apical processes of the retinal pigmented epithelium (RPE) [10, 19, 20]. Histologic studies in cat eyes have demonstrated that following retinal detachment, outer segment degeneration of rods and cones is rapid and precedes inner segment degeneration, suggesting IZ loss may precede EZ loss [21, 22]. Studies in a variety of retinal conditions also suggest that IZ disruption is associated with overlying EZ loss and visual acuity decline [23–26].

While extensive research has documented the importance of EZ loss as a metric in MacTel progression, the role of the IZ band has not been systematically explored in MacTel patients beyond small case studies [27–29]. Recently, in eyes with early MacTel, our group identified cone mosaic lesions on AOSLO corresponding to areas of qualitative IZ disruption in areas where the EZ was completely intact [29]. Considering the spatial and temporal relationships between IZ and EZ disruption observed in other degenerative retinal diseases, we sought to investigate the extent of IZ disruption and its spatial overlap with DCP telangiectasia across the entire disease spectrum of MacTel [23–26]. We hypothesized that IZ disruption could occur in at-risk or dysfunctional photoreceptors that later would go on to show loss of EZ visibility. Specifically, we predicted that IZ disruption would overlap better with DCP telangiectasia in earlier stages of MacTel, when EZ loss is still minimal. We also analyzed follow-up imaging to investigate longitudinal relationships between IZ and EZ disruption.

## Materials and methods

This observational cross-sectional study enrolled 23 patients (44 eyes) diagnosed with MacTel between March 2016 and July 2020 in the Department of Ophthalmology at Northwestern University in Chicago, Illinois. The study was approved by the Institutional Review Board of Northwestern University (IRB no. STU00200890) and conducted in accordance with the tenets of the Declaration of Helsinki and the regulations of the Health Insurance Portability and Accountability Act. Written informed consent was obtained from all participants.

Patients received baseline optical coherence tomography angiography (OCTA) imaging between August 2018 and July 2020. Five patients (ten eyes) underwent repeat OCTA imaging at 6-month follow-up. Further retrospective review revealed that one subject also received prior OCTA imaging two and half years before baseline imaging, with available imaging spanning three years total. We excluded eyes from the longitudinal analysis that received treatment as participants of an ongoing clinical trial.

Each patient underwent full ophthalmological exam. Best corrected Snellen visual acuity was assessed and converted to logarithm of the minimum angle of resolution (logMAR). Color fundus photography and fundus fluorescein angiography (FFA) were performed and MacTel was diagnosed based on the presence of characteristic funduscopic findings (perifoveal translucency, inner retinal crystals, non-tapering angled venules, and dilated capillaries) with typical FFA patterns (telangiectatic capillaries with late-phase hyperfluorescence). OCTA imaging was obtained for both eyes of each patient when possible. Using the corresponding SD-OCT scans from the OCTA instrument, eyes were staged using OCT-based criteria proposed by Chew et al., which considers presence and location of EZ loss and macular pigment deposits, presence of hyperreflective foci, and subretinal neovascularization (Chew EY, et al. IOVS 2019;60:ARVO E-Abstract 1335).

Demographic and clinical data were obtained from electronic medical records. Age, gender, race, and diagnosis of diabetes and systemic hypertension were recorded for each patient.

### Image acquisition

OCTA images were obtained using the RTVue-XR Avanti system (Optovue Inc., Fremont, California, USA) with split-spectrum amplitude-decorrelation angiography (SSADA) algorithm [30]. Two consecutive B-scans, each containing 304 A-scans, were captured over a 3 mm × 3 mm region (304 pixels × 304 pixels) centered on the fovea. The A-scan rate was 70,000 scans/s, using a light source centered on 840 nm and a bandwidth of 45 nm. Information about angiographic flow was extracted from both B-scans with the SSADA algorithm. 3D Projection artifact removal (3D-PAR) technology by Optovue was applied to the images before extraction for further analysis. Only images with a quality index (Q-score) of 7 or greater, signal strength index (SSI) of 55 or greater, and no significant motion or shadow artifacts were included.

### Image analysis

*En face* angiograms of the SCP and DCP were segmented using the built-in AngioVue Analytics software (version 2017.1.0.151) according to default parameters. The SCP was segmented from the internal limiting membrane (ILM) to 10 μm above the inner plexiform layer (IPL). The DCP was segmented from 10 μm above the IPL to 10 μm below the outer plexiform layer. To examine the EZ and IZ, an OCT slab containing both layers was manually segmented from the inner segment and outer segment junction to 15 μm above the retinal pigment epithelium (RPE). All images were reviewed, and any segmentation errors corrected manually.

Image analysis was performed in FIJI, a distribution of the program ImageJ [31]. DCP telangiectasias were identified as non-tapering and dilated vessels as we have previously described [16]. Circumscribed areas were not outlined freehand but instead using a partially automatic method, where local Phansalkar thresholding was used to isolate individual vessels from marked DCP telangiectasia areas. The binarized vessel map was processed using the ImageJ method Fill Holes (developed by Gabriel Landini, University of Birmingham, UK) to obtain circumscribed areas corresponding to DCP telangiectasia. Manual correction was performed as necessary to ensure all circumscribed areas were included. Because large vessel artifacts from the SCP can appear like DCP telangiectasia, SCP large vessels were identified via Max Entropy thresholding and superimposed onto DCP telangiectasia maps to distinguish artifact from true dilated vessels.

**Fig 1:**
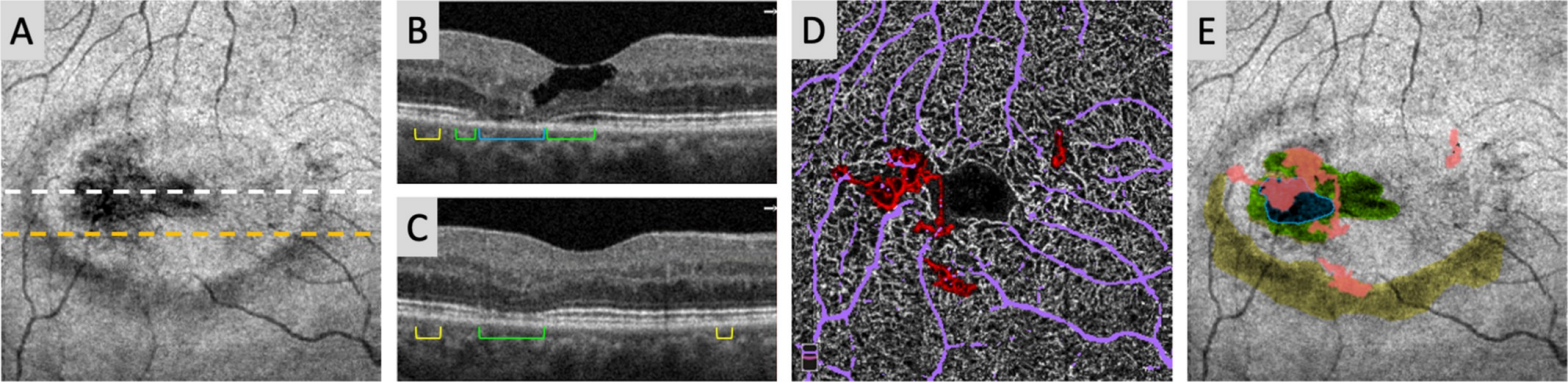
Identification and grading of DCP telangiectasia, IZ attenuation, IZ loss, and EZ loss in MacTel eyes. (A) Right eye of a 59-year old female with nonproliferative (OCT stage 3) MacTel. *En face* images of a manually defined OCT slab located between the inner segment-outer segment (IS/OS) junction and 15 μm above the RPE were used to identify areas of potential hyporeflectivity in the IZ and EZ layers and cross-referenced with the OCT cross-sectional B-scans (B–white dashed line; C–orange dashed line) to delineate areas of IZ attenuation (yellow), IZ loss (green), and EZ loss (blue; outlined for clarity). (D) Grading of DCP telangiectasia (red) and large superficial vessel overlay (lavender) in the same eye. (E) Overlap of circumscribed regions of DCP telangiectasia with IZ attenuation, IZ loss, and EZ loss areas described in (A).

Areas of IZ disruption were identified as regions of hyporeflectivity on *en face* OCT and characterized by extent of change confirmed on the cross-sectional B-scans, as shown in Fig 1. IZ attenuation areas were defined as present but hyporeflective IZ band with intact EZ band above. IZ loss was defined as absence of the IZ band, as confirmed by distinct IZ break or “buckling” of the intact overlying EZ band. EZ loss was defined as areas of EZ defect as previously described [11, 16]. Furthermore, we required that areas with EZ loss also have IZ loss.

Corresponding IZ attenuation, IZ loss, and EZ loss areas were traced for each eye using ImageJ’s freeform polygon tool from *en face* OCT. Due to the directly contiguous nature of regions of IZ attenuation, IZ loss, and EZ loss, we first traced a combined region containing all photoreceptor disruption (IZ attenuation + IZ loss + EZ loss areas). A second combined region containing IZ loss + EZ loss areas was then separately traced, followed by a final tracing of EZ loss area. To isolate IZ attenuation areas only, the IZ loss + EZ loss region was subtracted from the IZ attenuation + IZ loss + EZ loss region. IZ loss areas were similarly determined by subtracting the EZ loss region from the IZ loss + EZ loss region. To assess the intergrader reliability for IZ attenuation, IZ loss, and EZ loss regions, a random subset of 12 eyes was traced independently by a second grader (R.Z.).

Areas of DCP telangiectasia, IZ attenuation, IZ loss, and EZ loss were quantified as total number of pixels and converted to square millimeters (mm^2^). Percent overlap of DCP telangiectasia area with IZ attenuation, IZ loss, and EZ loss areas was determined as the number of overlapping pixels expressed as a percentage of the total DCP telangiectasia area.

**Fig 2:**
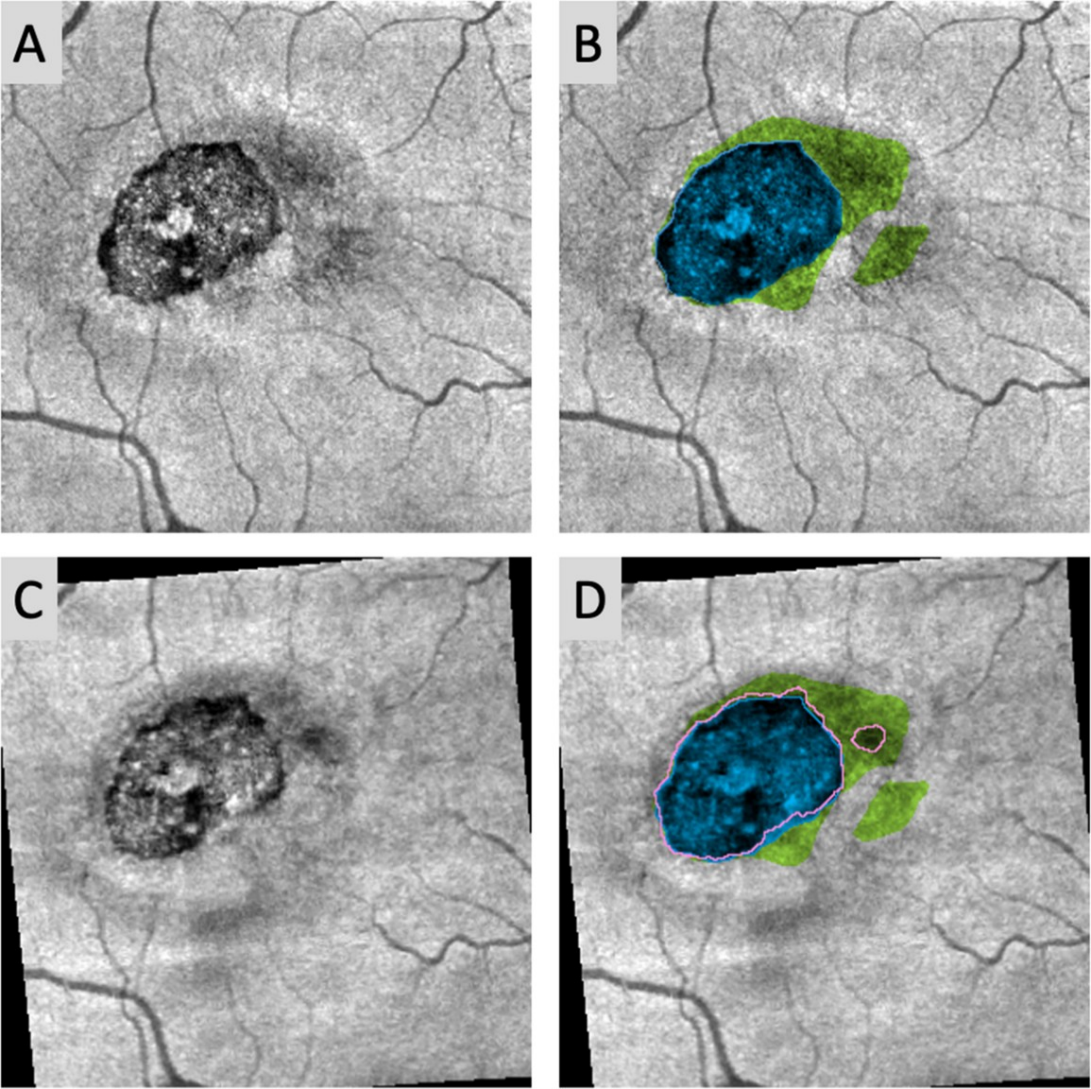
Determining predictive value of IZ loss for future EZ loss. (A) Left eye of a 49-year old with proliferative (OCT Stage 6) MacTel at baseline imaging. (B) Areas of IZ loss (green), and EZ loss (blue; outlined for clarity) at baseline. (C) Same eye imaged at 6-month follow-up, registered to align with (A). (D) EZ loss region at 6-month follow-up (pink outline) superimposed onto B. Note how the new circular area of EZ loss to the right of the main EZ loss area falls entirely within an area of previous IZ loss.

For patients who underwent longitudinal imaging, we sought to assess whether IZ loss could predict future EZ loss. The follow-up OCTA scans of the SCP were first aligned to the baseline SCP scans using bUnWarpJ, a plugin for elastic image registration [32]. The elastic transform determined from aligning the SCP was then applied to the regions of IZ loss and EZ loss. Areas corresponding to apparent new EZ loss were calculated by subtracting the baseline and follow-up EZ loss regions, and the extent of their overlap with previous IZ loss areas measured, as shown in Fig 2. Relative positive and negative predictive values were calculated as described by Hwang et al. [33]. Briefly, the positive predictive value (PPV) describes the likelihood of previous IZ loss to predict new EZ loss, while the negative predictive value (NPV) describes the likelihood of areas without IZ loss to remain without new EZ loss. The relative positive (rPPV) and negative predictive values (rNPV) have been normalized to represent the PPV and NPV relative to that which would be expected by random chance; hence an rPPV or rNPV of 1 would suggest predictive value no better than chance.

### Statistical analysis

Statistical analyses were performed using IBM SPSS statistics version 26 (IBM SPSS Statistics; IBM Corporation, Armonk, MY, USA). Two-tailed P-values of <0.05 were considered significant for all tests. To assess intergrader reliability of IZ attenuation, IZ loss, and EZ loss area determination, intraclass correlation coefficients (ICCs) were calculated for a randomly selected sample of 12 eyes evaluated independently by two graders (J.X.O. and R.Z).

Due to the ordinal nature of the Chew et al. MacTel staging, we chose the Spearman correlation test to study associations involving MacTel stage, to avoid assuming linearity between increasing stages. Spearman correlations were calculated to assess the preliminary relationships of OCT-based MacTel stage with the areas and percentage overlaps of DCP telangiectasia, IZ attenuation, IZ loss, and EZ loss.

We then pooled patients by stage into groups representing early (Stage 0–2), moderate (Stage 3–5), and advanced (Stage 6) MacTel. To study inter-group differences, we used generalized estimating equations (GEE) adjusting for two eyes from the same patient and determined Wald chi-square statistics to assess the effect of pooled MacTel stage on DCP telangiectasia, IZ, and EZ parameters. Significant parameters were further evaluated using post-hoc pairwise tests with Bonferroni correction for multiple comparisons. We also used GEEs to evaluate the association of the DCP telangiectasia, IZ, and EZ parameters with diagnosis of diabetes or hypertension.

## Results

Of the 44 eyes (23 patients) initially imaged, 9 were excluded due to insufficient OCTA image quality, yielding a final sample of 35 eyes (21 patients). Patient demographic and clinical characteristics are summarized in Table 1. Eyes were distributed between early, moderate, and advanced MacTel stages, with 12 eyes meeting criteria for stages 0–2, 10 eyes meeting stage 3–5 criteria, and 13 eyes meeting stage 6 criteria. We evaluated intergrader reliability in a random subset of 12 eyes graded by a second masked grader by calculating ICC for IZ attenuation (ICC = 0.703; *p* = 0.004), IZ loss (ICC = 0.806; *p* < 0.001), and EZ loss areas (ICC = 0.970; *p* < 0.001). All 35 eyes exhibited areas of apparent IZ attenuation, while 33 of the 35 eyes (94.3%) showed evidence of IZ loss and 29 of the 35 eyes (82.9%) showed EZ loss. Overall, areas of IZ attenuation and loss were located around areas of EZ loss (Fig 1).

**Table 1:** Demographics, clinical characteristics, and MacTel staging of study patients.

Characteristic	Mean ± SD (range)
Subjects	21
Eyes	35
Age (years)	58.2 ± 8.8 (43–72)
Sex	
Male	8
Female	13
Laterality	
OD	18
OS	17
Visual acuity	
LogMAR	0.189 ± 0.223 (-0.187–0.903)
Diabetes	7
Hypertension	6
OCT stage	
0	6
1	3
2	3
3	3
4	7
5	0
6	13

Abbreviations: LogMAR = logarithm of the minimum angle of resolution; OCT = optical coherence tomography; OCTA = optical coherence tomography angiography; OD = right eye; OS = left eye; SD = standard deviation.

Correlation of MacTel severity stage with DCP telangiectasia, IZ, and EZ parameters are summarized in Table 2. Area of IZ loss correlated significantly with advancing MacTel severity stage (Spearman rank correlation, *R*s = 0.742, *p* < 0.001), while IZ attenuation showed a trend of positive correlation with severity (*R*s = 0.341, *p* = 0.045). In contrast, the percentage of overlap between DCP telangiectasia and IZ attenuation decreased with increasing MacTel severity (*R*s = -0.667, *p* < 0.001), while overlap with IZ loss increased (*R*s = 0.407, *p* = 0.015). DCP telangiectasia, EZ loss, and percent overlap between telangiectasia and EZ loss correlated most strongly with MacTel severity stage (*R*s = 0.881–0.940, *p* < 0.001), as we have previously shown [16].

**Table 2:** Correlations between OCT-based MacTel stage and MacTel imaging parameters: DCP telangiectasia, photoreceptor defect areas and their overlap.

Parameter	Spearman *R-*value	Spearman *p*-value
Area (mm^2^)		
DCP telangiectasia	0.881	*p* < 0.001
IZ attenuation	0.341	*p* = 0.045
IZ loss	0.742	*p* < 0.001
EZ loss	0.940	*p* < 0.001
Overlap with DCP telangiectasia (%)		
IZ attenuation	-0.667	*p* < 0.001
IZ loss	0.407	*p* = 0.015
EZ loss	0.899	*p* < 0.001

Abbreviations: DCP = deep capillary plexus; IZ = interdigitation zone; EZ = ellipsoid zone.

**Fig 3:**
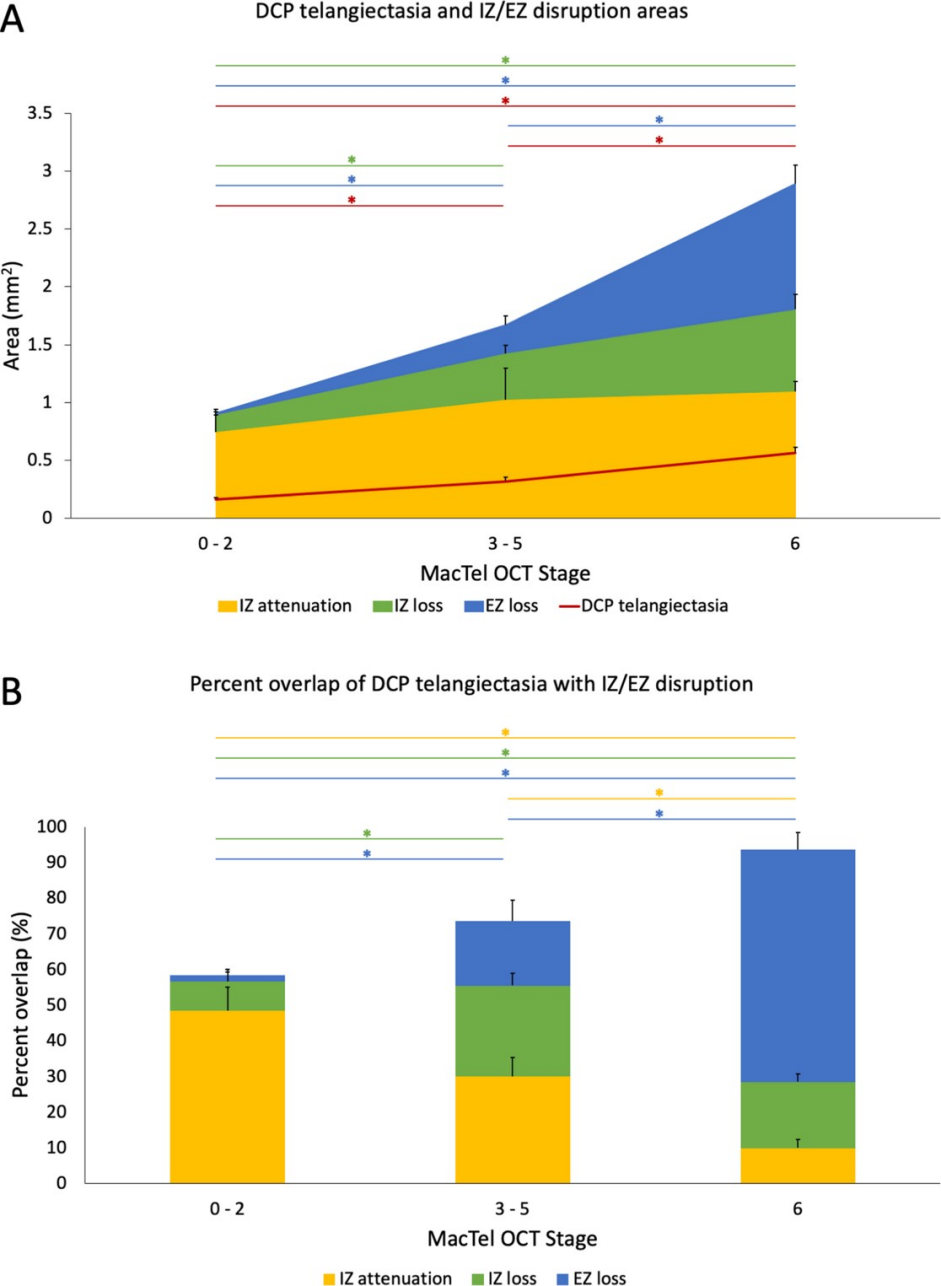
Areas and overlaps of DCP telangiectasia with photoreceptor defect parameters across MacTel OCT stage groups. MacTel eyes were classified by pooling OCT-based severity stages into early (Stages 0–2), moderate (Stages 3–5), and advanced (Stage 6). (A) Total area of IZ attenuation (yellow), IZ loss (green), EZ loss (blue), and DCP telangiectasia (red) with MacTel OCT stage. (B) Percent overlap of DCP telangiectasia with IZ attenuation (yellow), IZ loss (green), and EZ loss (blue) with MacTel stage. Generalized estimating equations were used to adjust for the contribution of two eyes from the same patient and differences between groups were compared using post-hoc pairwise tests with Bonferroni correction. Error bars represent 1 SE. Asterisks indicate statistically significant differences (*p* < 0.05) between groups.

Using GEEs adjusting for the effect of multiple eyes of the same patient, we also compared DCP telangiectasia, IZ, and EZ parameters between early (Stage 0–2), moderate (Stage 3–5), and advanced (Stage 6) MacTel groups (Table 3). Fig 3 shows the changes in total areas (Fig 3A) and percent overlaps (Fig 3B) of DCP telangiectasia, IZ attenuation, IZ loss, and EZ loss areas with increasing MacTel stage and the results of *post-hoc* pairwise comparisons between disease groups. In summary, IZ attenuation area showed no significant differences between groups, while IZ loss area was significantly higher in both moderate and advanced MacTel compared to early (both *p* < 0.001). Both EZ loss area (*p* = 0.004 through < 0.001) and DCP telangiectasia area (*p* = 0.002 through < 0.001) increased with MacTel severity and significantly differed for all comparisons between groups.

**Table 3:** By-group analysis of DCP telangiectasia area, photoreceptor defect areas and their overlap percentages with DCP telangiectasia by pooled OCT-based MacTel stages.

Parameter,	Stages 0–2	Stages 3–5	Stage 6	GEE (Wald χ^2^)
Mean ± SD	(n = 12)	(n = 10)	(n = 13)	
Area (mm^2^)				
DCP telangiectasia	0.160 ± 0.059	0.313 ± 0.123	0.561 ± 0.190	*p* < 0.001
IZ attenuation	0.745 ± 0.500	1.024 ± 0.856	1.095 ± 0.321	*p* = 0.027
IZ loss	0.150 ± 0.149	0.401 ± 0.221	0.711 ± 0.464	*p* < 0.001
EZ loss	0.016 ± 0.031	0.247 ± 0.237	1.091 ± 0.560	*p* < 0.001
Overlap with DCP telangiectasia (%)				
IZ attenuation	48.55 ± 22.68	30.09 ± 16.61	10.06 ± 8.36	*p* < 0.001
IZ loss	8.21 ± 11.64	25.49 ± 10.55	18.44 ± 8.06	*p* < 0.001
EZ loss	1.60 ± 3.73	17.94 ± 18.82	65.12 ± 17.79	*p* < 0.001

Abbreviations: SD = standard deviation; DCP = deep capillary plexus; IZ = interdigitation zone; EZ = ellipsoid zone.

Percent overlap between DCP telangiectasia and IZ attenuation was significantly higher in early vs. advanced MacTel (*p* < 0.001), as well as in moderate vs. advanced (*p* < 0.001). Percent overlap of DCP telangiectasia with IZ loss was highest in the moderate group and significantly differed in early vs. moderate and advanced MacTel (both *p* < 0.001). Percent overlap with EZ loss was highest in the advanced group and significantly differed for all comparisons between the three groups (*p* = 0.012 through < 0.001). We found no significant associations between prevalence of diabetes (*p* = 0.171 to 0.726) or hypertension (*p* = 0.396 to 0.980) with any DCP telangiectasia, IZ and EZ parameters.

Follow-up imaging at 6 months was available for 10 eyes, of which 2 eyes were excluded due to poor image quality and 2 excluded who were enrolled in an experimental study, leaving a final sample of 6 eyes (Stage 1: 1 eye, Stage 2: 1 eye, Stage 4: 1 eye, Stage 6: 3 eyes) with longitudinal data. Because EZ loss area is widely accepted as a marker of MacTel progression, we focused on EZ changes and their relationship to IZ loss, which showed higher inter-rater reliability than IZ attenuation [28]. Overall, the boundaries of EZ loss area showed dynamic changes over time, with 5 eyes exhibiting areas of new EZ loss (0.052 mm^2^ ± 0.022 mm^2^, mean ± SD) and all 6 eyes exhibiting areas of EZ recovery (0.045 mm^2^ ± 0.023 mm^2^). Before normalization, we found that for new EZ loss, baseline IZ loss showed low PPV (mean PPV = 8.9% ± 5.3%), while absence of IZ loss showed high NPV (mean NPV = 99.9% ± 0.1%). After normalizing predictive values relative to random chance, we found that baseline IZ loss was substantially more likely than chance to predict new EZ loss at 6 months (mean rPPV = 15.0 ± 8.1), while absence of IZ loss at baseline was no better than chance at predicting future EZ integrity (mean rNPV = 1.0 ± 0.0).

**Fig 4:**
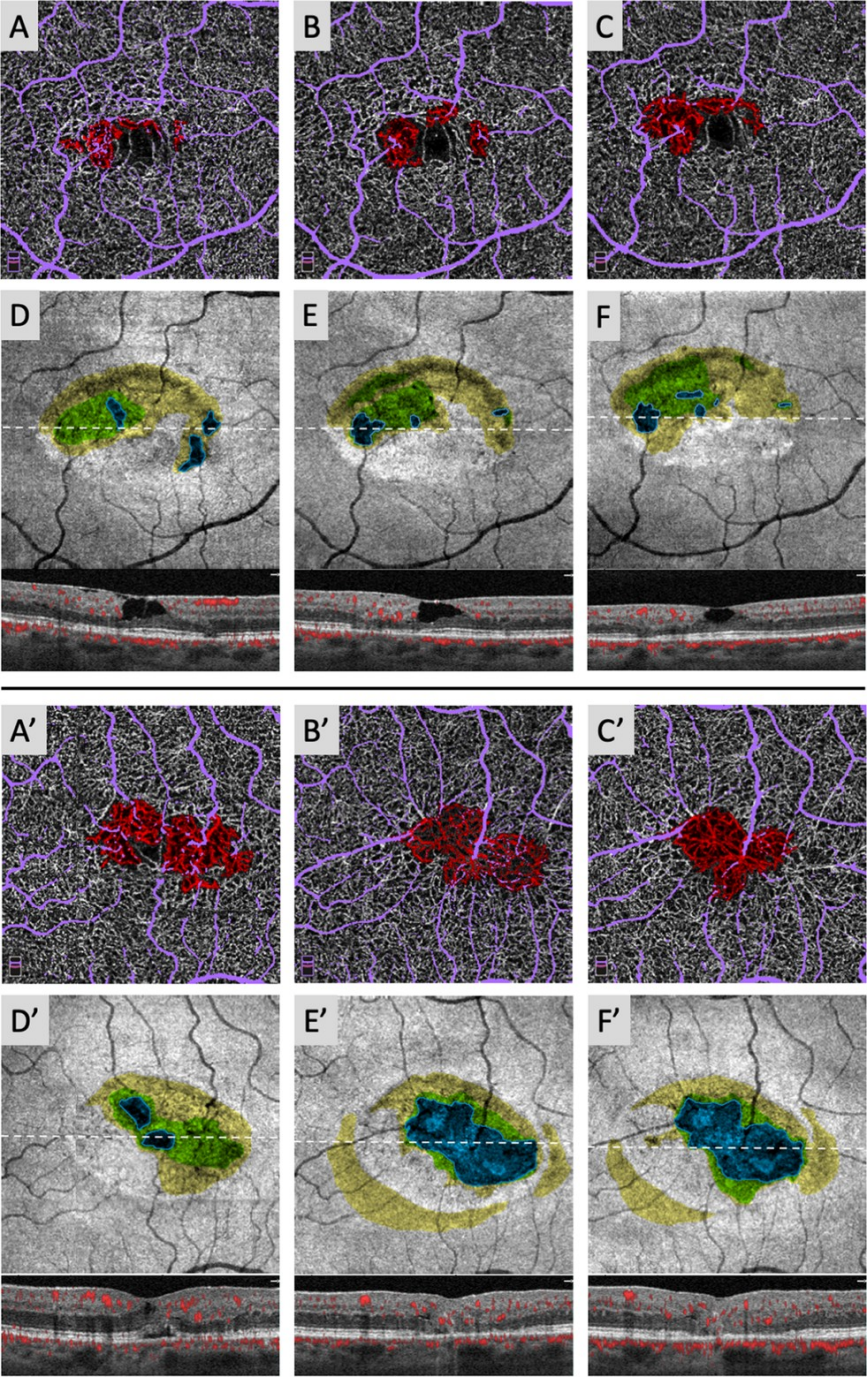
Areas of IZ loss qualitatively predict future EZ loss in long-term follow-up. Right eye (A–F) and left eye (A’–F’) of 56-year old male with nonproliferative (Right eye: OCT Stage 2; left eye: OCT Stage 3) MacTel at initial imaging (left column). OCTA imaging of DCP telangiectasia (red) and large superficial vessel overlay (lavender) shows dynamic changes in telangiectasia boundaries in both eyes (A–C and A’–C’), with substantial superficial vessel structural change in the left eye at 2.5 years (B’). Areas of IZ attenuation (yellow), IZ loss (green), and EZ loss (blue; outlined for clarity) on the OCT slab show dynamic changes in the right eye (D–F), while the left eye (D’–F’) shows overall coalescence and enlargement of EZ loss, expanding within and ultimately replacing areas of IZ loss.

We also qualitatively assessed longer-term IZ and EZ changes in one subject with three years of longitudinal follow-up, shown in Fig 4. Dynamic changes in areas of DCP telangiectasia, IZ attenuation, IZ loss, and EZ loss can be seen in both eyes over time, with similar patterns of IZ loss preceding EZ loss. Notably, the left eye showed two small areas of central EZ loss surrounded by a large zone of IZ loss at the earliest imaging timepoint that eventually progressed to a large area of EZ loss over 2.5 years. This large EZ loss area remained stable in size a further 6 months afterwards. The final timepoint for the left eye of this subject was volume-rendered to better visualize the relationships between DCP telangiectasia, diving superficial vessels, IZ loss, and EZ loss (S1 Video).

## Discussion

In this study, we sought to characterize IZ abnormalities and their relationship to accepted markers of photoreceptor disruption (EZ loss) and vascular changes (DCP telangiectasia) in MacTel. In Stage 0 eyes, which lack EZ disruption, areas of IZ attenuation were identified in 6 of the 6 eyes, while IZ loss was present in 4 eyes. When IZ and EZ disruption lesions co-existed, EZ loss was generally located in the center of a larger zone of IZ loss (Figs 1, 2 and 4). Overall, we found that the extent of overlap between DCP telangiectasia and photoreceptor disruption showed an interesting pattern as MacTel severity increased. Cross-sectionally, we observed that DCP telangiectasias tended to overlie areas with IZ-only attenuation or loss in early to moderate MacTel, while in advanced MacTel, DCP telangiectasia overlapped with areas of combined EZ and IZ disruption (Fig 3). These results are consistent with an overall model of IZ disruption occurring in the early stages, which then progresses to combined IZ and EZ changes in the later stages (Tables 2 and 3 and Fig 3). Our results are also consistent with qualitative data from previous small studies of MacTel eyes from our group and others, which found that areas of IZ disruption colocalize with hyporeflective lesions on AOSLO in zones where the EZ is still intact [27, 29].

Longitudinal imaging demonstrated that preexisting IZ loss was relatively predictive of subsequent EZ loss, while absence of IZ loss was not necessarily predictive of EZ preservation (Figs 2 and 4). New EZ loss almost always emerged in areas with previous IZ loss; conversely, however, most areas of IZ loss did not convert to EZ loss within 6 months. We realize that this duration of follow-up is relatively short in the course of a disease that is overall slowly progressive. Our observations resemble patterns seen in patients with other forms of photoreceptor damage including retinitis pigmentosa, a genetic disease that causes loss of both rods and cones, as well as acute posterior multifocal placoid pigment epitheliopathy, an acquired inflammatory chorioretinopathy; and in patients recovering from macular hole surgery [23, 25, 26, 34]. Within the brief 6 month follow-up window, we saw dynamic changes with areas of progression as well as recovery around the margins of pre-existing EZ loss (Figs 2 and 4). However, despite the apparently reversible nature of these short-term changes, Gaudric et al. recently showed that MacTel eyes with EZ loss at baseline experience significant total increases in EZ loss area over a 3-year follow-up period, consistent with an overall long-term progression in EZ loss area [35]. Our findings of a large area of IZ loss progressing to stable EZ loss in our patient with three-year follow-up suggest that while the EZ may show dynamic changes in the short-term, areas of preexisting IZ disruption may predict long-term EZ loss (Fig 4).

Taken together, the data support the hypothesis that IZ disruption could be a potential indicator of “photoreceptors at risk”—photoreceptor areas with subtle pathological abnormalities that precede EZ disruption—with these areas tightly corresponding with overlying vascular abnormalities in early MacTel. Several physiologic factors may contribute to the apparent earlier onset of IZ disruption compared to EZ disruption. The cone outer segments that make up the IZ band experience significant daily recycling and regeneration, a process that maintains maximum sensitivity of their light-capturing membranes, which initiate the visual cascade [36, 37]. The high turnover of the cone outer segments may make the IZ layer more sensitive to early photoreceptor dysfunction than the EZ, which is comprised of inner segments primarily containing cellular organelles [37]. *In vitro* studies of various species have also suggested that in addition to their regulatory and supportive roles, Müller cells may contribute to cone cell renewal via phagocytosis and chromophore recycling of the cone outer segment membranes [4, 38, 39]. The central role of Müller cell loss in MacTel has been demonstrated both histologically in human MacTel eyes as well as in mouse models, where Müller cell ablation produces photoreceptor loss and vascular changes that resemble MacTel [3, 5, 40]. Considering the extensive physiologic cycling of the outer segment membranes and the important role of Müller cells in cone outer segment turnover, we would predict the IZ layer to show earlier signs of photoreceptor dysfunction in MacTel eyes, as our data suggest [21].

Although we found IZ attenuation and IZ loss underlying much of the DCP telangiectasia in early and moderate MacTel eyes, approximately 40% of DCP telangiectasia area in the Stage 0–2 MacTel eyes showed no discernible IZ or EZ disruption. The current neurodegenerative model of MacTel would suggest that vascular changes are secondary to underlying photoreceptor dysfunction [3, 40]. These isolated telangiectatic areas may represent an earlier, “pre-IZ” stage of photoreceptor dysfunction that cannot be assessed on SD-OCT but may be visualized on imaging modalities like AOSLO [29]. Additionally, while both SD-OCT and AOSLO ultimately assess structural integrity of the photoreceptors, functional disruption will most likely precede observable structural damage. We previously hypothesized that subclinical photoreceptor dysfunction may disrupt the normal anti-angiogenic signaling by photoreceptors in MacTel, which is consistent with observations of subretinal neovascularization originating from DCP telangiectasias in longitudinal follow-up of MacTel eyes [11, 16, 41]. A pro-angiogenic microenvironment resulting from diminished photoreceptor function may be sufficient to promote dilation of DCP vessels and thus telangiectasia formation, even before the appearance of structural photoreceptor changes. Finally, we acknowledge that vessel parameters, especially in the deep capillary layers, are susceptible to OCTA imaging artifacts and scan quality, which may cause some areas of non-dilated vessels to artifactually appear like DCP telangiectasia [42].

One potential caveat of our study is the inherent subjectivity associated with assessment of the IZ layer, due to baseline variations and imaging conditions. To mitigate potential subjectivity, we based our assessment of IZ attenuation and loss on both *en face* and cross-sectional images. Using these criteria, we found reasonable intergrader reliability for IZ attenuation and IZ loss, with IZ loss overall appearing to be a more reliable metric. In the future, we believe reliability could be further improved through automated quantification methods or image quality improvement by using multiple scans. It is possible that a small fraction of our subjects had a physiologic IZ area smaller than the 9 mm^2^ scan area imaged on SD-OCT, which could overestimate their extent of IZ attenuation [43]. Qualitative studies have also suggested that IZ reflectivity is influenced by the angle at which the eye is imaged [44]. Due to these considerations, we believe IZ loss may represent a more consistent measure of IZ disruption than IZ attenuation. While IZ hyporeflectivity (and thus IZ attenuation) could be relatively subjective due to the above factors, we believe the IZ loss parameter is more robust. Notably, we required the presence of overlying EZ deformation or a distinct IZ band break in the cross-sectional view for this parameter and confirmed these disruptions further against the presence of adjacent zones of intact IZ band in the same image.

Other limitations of our study include the relatively short duration of follow-up and the use of manual outlining of DCP telangiectasia, which is subject to individual interpretation as well as OCTA scan quality [16]. We also did not consider status of the external limiting membrane, which has been demonstrated to have significant value in predicting future photoreceptor recovery in conditions like macular hole surgery [26, 45].

In conclusion, this study builds on our previous hypothesis of “photoreceptors at risk” by characterizing IZ disruption as a potential precursor to EZ loss in MacTel. We found evidence of IZ attenuation or loss in early MacTel eyes, even in those with no evidence of EZ disruption. These areas of IZ disruption show greater overlap with DCP telangiectasia in early and moderate MacTel, at a stage when EZ loss borders DCP telangiectasia [16]. Furthermore, based on longitudinal imaging data, we show that IZ loss is not only generally correlated with MacTel severity, but also predictive of future EZ loss. Overall, our findings suggest that areas of IZ loss and their overlap with DCP telangiectasia suggest subclinical (“pre-EZ”) photoreceptor dysfunction in MacTel, as well as areas at higher risk of future EZ loss. Future longitudinal studies to investigate the relationships between IZ disruption, EZ loss, and corresponding functional vision changes are warranted.

## Supporting information

S1 Video Volume-rendered left eye of a 56-year old male with proliferative (OCT stage 6) MacTel at the 3-year timepoint from Fig 4. DCP telangiectasias (red) show coalescence towards an area of central neovascularization, which overlies a large region of EZ loss (blue) surrounded by a border of IZ loss (green). Diving superficial vessels are indicated in lavender.
(MOV)

S1 Dataset Raw demographic, clinical and optical coherence tomography/angiography data. (XLSX)
